# Modulation of plant defense responses to herbivores by simultaneous recognition of different herbivore-associated elicitors in rice

**DOI:** 10.1038/srep32537

**Published:** 2016-09-01

**Authors:** Tomonori Shinya, Yuko Hojo, Yoshitake Desaki, John T. Christeller, Kazunori Okada, Naoto Shibuya, Ivan Galis

**Affiliations:** 1 Institute of Plant Science and Resources, Okayama University, Kurashiki, Okayama 710-0046, Japan; 2 Department of Life Sciences, School of Agriculture, Meiji University, Kawasaki, Kanagawa 214-8571, Japan; 3 The New Zealand Institute for Plant & Food Research, Palmerston North 4442, New Zealand; 4 Biotechnology Research Center, The University of Tokyo, Tokyo 113-8657, Japan

## Abstract

Induced plant defense responses against insect herbivores are triggered by wounding and/or perception of herbivore elicitors from their oral secretions (OS) and/or saliva. In this study, we analyzed OS isolated from two rice chewing herbivores, *Mythimna loreyi* and *Parnara guttata*. Both types of crude OS had substantial elicitor activity in rice cell system that allowed rapid detection of early and late defense responses, i.e. accumulation of reactive oxygen species (ROS) and defense secondary metabolites, respectively. While the OS from *M. loreyi* contained large amounts of previously reported insect elicitors, fatty acid-amino acid conjugates (FACs), the elicitor-active *P. guttata*’s OS contained no detectable FACs. Subsequently, elicitor activity associated with the high molecular mass fraction in OS of both herbivores was identified, and shown to promote ROS and metabolite accumulations in rice cells. Notably, the application of *N*-linolenoyl-Gln (FAC) alone had only negligible elicitor activity in rice cells; however, the activity of isolated elicitor fraction was substantially promoted by this FAC. Our results reveal that plants integrate various independent signals associated with their insect attackers to modulate their defense responses and reach maximal fitness in nature.

Insect herbivores are highly mobile and thus represent an unpredictable threat for plants. They vigorously attack leaves, stems, roots, reproductive organs and fruits, inflicting mechanical damage associated with their feeding strategy (e.g., chewing, piercing, sucking). Experiments with robotic mechanical wounding (MecWorm) showed that repeated wounding alone can mimic herbivore damage in plants^1^. Mechanical wounding is known to strongly elicit jasmonic acid (JA) biosynthesis, and JA signaling that is, in many cases, further modified by accessory signals such as elicitors from diverse insects^2^,^3^. The insect-specific molecules are thus believed to inform plants about their attackers, which may lead to better defense, especially when such signals are associated with mechanical stress^4^,^5^.

Insect-specific elicitors in plants include OS-, saliva-, and oviposition-associated factors. These elicitors can be small compounds, such as FACs (fatty acid-amino acid conjugates), inceptins, caeliferins, bruchins, as well as much larger molecules, such as the enzyme glucose oxidase^6^,^7^. Besides their mass, insect elicitors may differ in origin and/or molecular function, such as herbivore-associated elicitors (HAEs) derived from insects, damage-associated molecular patterns (DAMPs) from plants, effectors, and microbes (e.g., insect symbionts)^6^,^8^. The FACs are common lepidopteran insect HAEs that appear in large quantities in the caterpillar’s regurgitate during feeding^9^,^10^. Because of their wide distribution and high specificity to insect feeding, many plants evolved the means to recognize FACs as specific elicitors of defense (e.g., maize, eggplant, tobacco). The species-wide evolutionary conservation of FACs, which virtually betray herbivores to plants, has been attributed to their essential role in the amino acid metabolism in some insects^11^. Hydrogen peroxide (H_2_O_2_) and other reactive oxygen species (ROS) are well-known modulators and/or activators of plant defense^12^,^13^. Here, insect salivary glucose oxidase can catalyze the production of H_2_O_2_ from plant glucose at the feeding sites. Inceptin peptides are fragments of ubiquitously occurring plant chloroplastic enzyme, ATPase, that is converted to plant elicitors by digestive proteases from the insects^14^.

In contrast to the steadily growing list of insect-derived elicitors, their intrinsic receptors remain unknown. One exception may be the high-affinity binding protein for volicitin (an FAC) found in maize (*Zea mays*) plasma membranes^15^. However, the putative receptor gene has not been identified to date. After perception, wounding and elicitors activate intracellular signaling such as plant stress hormones, MAPK cascade, and cause cytosolic [Ca^2+^] fluctuations^5^,^16^. Subsequently, plants respond to herbivores by direct defense and/or release volatile organic compounds (VOCs) that attract natural enemies of herbivores (termed as indirect defense)^4^,^17^.

As examples of direct defense, most plants produce proteinase inhibitors and wide range of secondary metabolites, such as alkaloids, terpenes, cyanogenic glucosides, and phenolics. For example, nicotine alkaloids, diterpene glucosides and phenolamides (PAs) are strongly induced in the wild tobacco (*Nicotiana attenuata*) plants by herbivore feeding, which makes them highly toxic to most of the existing herbivores^18^. While nicotine production is quite specific to *Nicotiana* species, other metabolites such as phenolamides (PAs) are more common in plants^19^,^20^,^21^. Notably, the suppression of PAs, *p-*coumaroylputrescine (CoP), caffeoylputrescine (CP) and feruloylputrescine (FP) by down-regulating the key transcriptional regulator MYB8 significantly compromised the *N. attenuata* defense against chewing herbivores^22^. Recently, the accumulation and defense function of CoP and FP in rice against sucking insects has also been reported^23^,^24^.

Considering the vast diversity of plants and insects, identification of insect elicitors and their receptors requires establishment of new models suited for each interaction. Succinct metabolic responses to herbivores^23^, large variety of natural pests, and multitude of genetic resources indicated rice as prime model for the study of plant-insect interactions in monocots. In addition to PAs, rice plants accumulate momilactone diterpenes^25^, proteinase inhibitors^26^, and emit many VOCs in response to herbivore attack^27^. Specialist herbivores such as rice skippers (*Parnara guttata*; Lepidoptera: Hesperiidae), rice semiloopers (*Naranga aenescens*; Lepidoptera: Noctuidae), and rice leaffolders (*Cnaphalocrocis medinalis*; Lepidoptera: Pyralidae) are abundant in rice paddies^28^,^29^. In addition, monocot generalists such as Loreyi armyworms (*Mythimna loreyi*; Lepidoptera: Noctuidae) or lawn armyworms (*Spodoptera mauritia*; Lepidoptera: Noctuidae) occasionally feed on rice plants^28^,^29^. Planthoppers, including rice brown planthopper (*Nilaparvata lugens*; Hemiptera: Delphacidae), which is an economically important vector of rice ragged stunt (RRSV) and rice grassy stunt (RGSV) viruses, provide suitable sucking/piercing models for experiments^29^,^30^. Finally, rice stem borers offer yet different mode of insect attack in rice stem^29^,^31^. Altogether, rice and its resources can be used to address many important questions in the rapidly developing field of plant-insect interactions.

However, even in well-established plant models, experiments with mobile insects remain challenging. Direct feeding on plants is less convenient for high throughput experiments due to its low reproducibility and demand for insect/damage-free plant material to avoid systemic signaling and memory effects in plants^32^,^33^. At this point, cultivated rice cells may be of use, following the well-established examples of monitoring the activity of many microbial elicitors, based on the reproducible changes in secondary metabolism, defense gene expression, and ROS production in treated cells^34^,^35^. Importantly, elicitors and/or purified fractions can be applied to cells directly without excessive wounding, normally required for penetration of plant cuticle, a protective film covering the epidermis of leaves.

In the initial study with rice cells, we focused on chewing generalist herbivore *M. loreyi*, and examined its OS-associated elicitor activities. This OS contained several typical FACs, initially thought to be the major elicitors. However, subsequent bioassays and fractionation studies revealed that some additional factors, such as the high molecular mass elicitor(s) (HME) fraction largely contributed to the overall activity of *M. loreyi* OS. This HME-containing fraction strongly activated production of reactive oxygen species (ROS) and promoted phytoalexin accumulations in rice cells. Accentuating the importance of the non-FAC insect elicitor(s) in rice signaling, the OS from *P. guttata* that naturally lacked any FACs also elicited profound metabolic changes in rice cell system.

## Results

### Establishment of *M. loreyi*-rice experimental system

**Figure 1:**
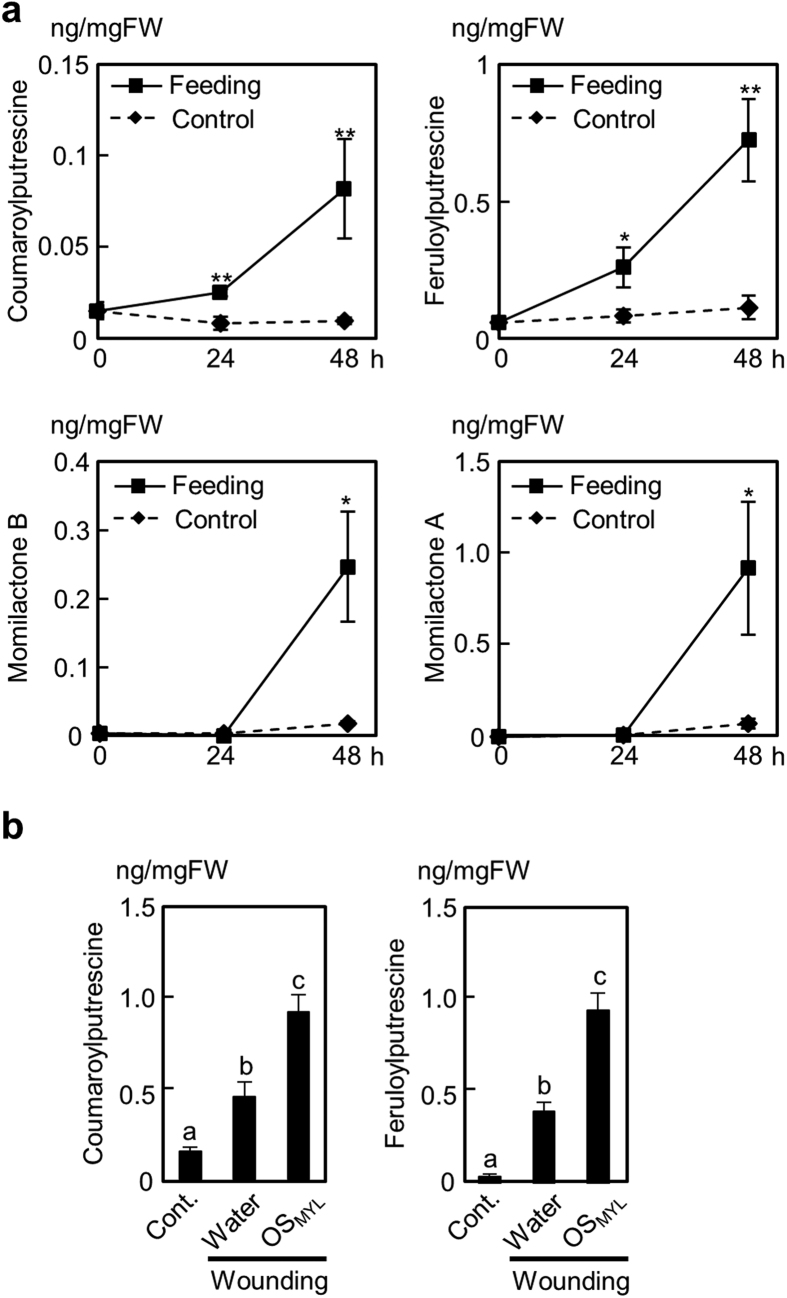
Metabolite accumulation induced by *M. loreyi* feeding and oral secretions in rice leaves. (**a**) *M. loreyi* feeding-induced metabolite accumulation in rice was determined by LC-MS/MS after 0, 1 and 2 d herbivore exposure. Data (n = 4–5) are shown as means ± SE, and asterisks show statistically significant differences between treated and control samples at each time point determined by Student t-test (**P < 0.01; *P < 0.05). (**b**) Rice leaves were wounded with a fabric pattern wheel, and immediately treated with 20 μL of water, or 20 μL of *M. loreyi* oral secretions (OS_MYL_, 3-fold dilution). Metabolite levels were measured 24 h after plant treatments. Data (n = 4–5) are shown as means ± SE, and statistical differences were analyzed by ANOVA followed by Tukey HSD test (P < 0.05).

Larvae of *M. loreyi* (abbreviated as “MYL” hereafter) are generalist pests feeding on grasses including rice plants^31^,^36^,^37^,^38^, providing a suitable model for identification and purification of insect elicitors active in rice. We first examined the metabolic responses elicited by MYL larvae in the intact rice leaves. MYL feeding significantly promoted the accumulation of two previously reported phenolamides, *p-*coumaroylputrescine (CoP) and feruloylputrescine (FP) (Fig. 1a), and additionally, it elicited two diterpene phytoalexins, momilactone A (MoA) and momilactone B (MoB) in rice leaves. This was in contrast with the previous report on low and inconsistent elicitation of MoA and MoB in rice leaves by two other chewing herbivores, *S. mauritia* and *P. guttata*^23^,^24^.

Next, herbivory mimic system consisting of application of MYL OS (OS_MYL_) to mechanically wounded rice leaves was used to examine a direct contribution of OS in defense elicitation. Compared to mechanical wounds treated with water, CoP and FP accumulations were significantly higher after addition of diluted OS_MYL_ to the leaves (Fig. 1b). However, the accumulation of rice diterpene phytoalexins, MoA and MoB was not observed in this system at 24 h time point (data not shown). Subsequently, we chose PAs (CoP and FP) as the main guide compounds for tracing OS_MYL_-associated elicitor activities in rice cell system. Although MYL was used as primary source of the elicitor-active OS, we also included a specialist insect, rice skipper *P. guttata*^23^ (abbreviated as “PAG” hereafter) in our current study. We assumed that in adaptive evolution, the insects with different levels of specialization to their host plants might have evolved somewhat different spectrum of elicitor and/or effector activities in the OS.

### Response of rice cells to OS_MYL_ and OS_PAG_

**Figure 2:**
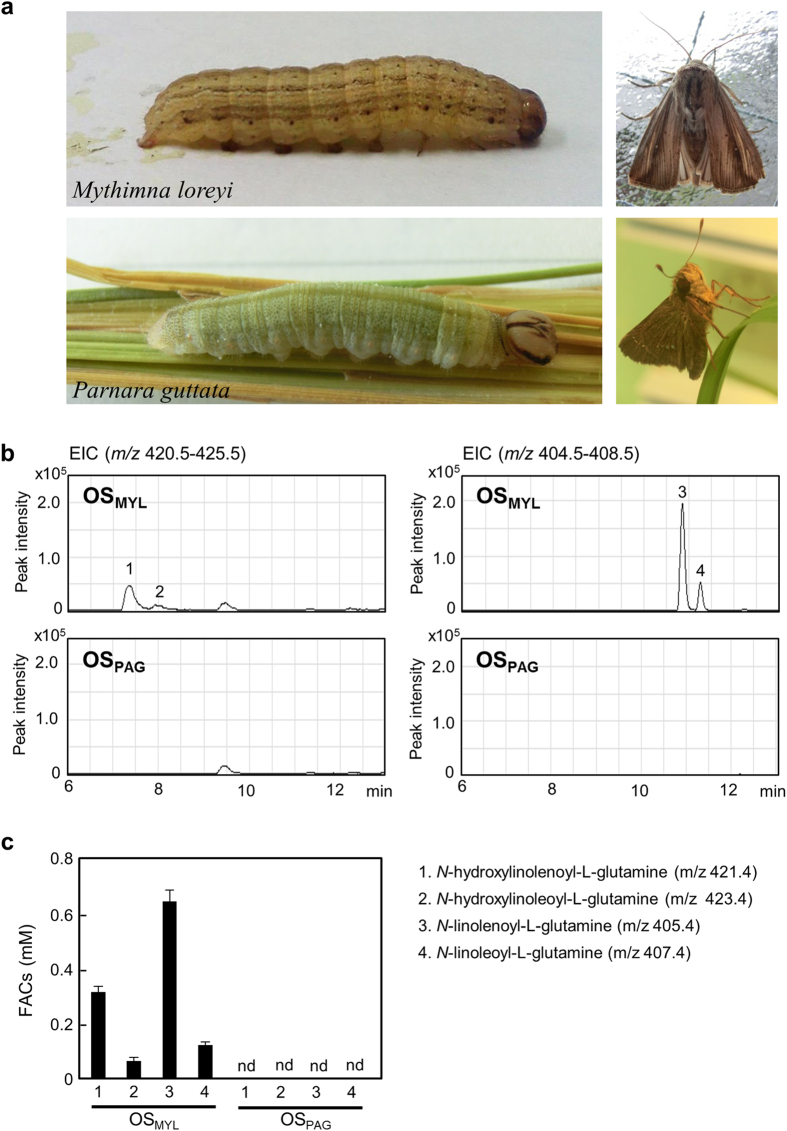
FAC composition and content in oral secretions from *M. loreyi* and *P. guttata.* (**a**) Caterpillars (left) and adults (right) of *M. loreyi* (OS_MYL_) and *P. guttata* used in experiments. (**b**) FAC composition in oral secretions from *M. loreyi* (OS_MYL_) and *P. guttata* (OS_PAG_) determined by LC-MS operating in negative scan MS mode (*m/z* range 150–1000); typical extracted ion chromatograms (EICs) of similarly diluted OS_MYL_ and OS_PAG_ samples are shown at unified *y-*axes scales for direct comparison. (**c**) The FACs in individual isolates of OS_MYL_ and OS_PAG_ were determined as above and total FAC concentration in each batch of OS were estimated by pooling individual FAC peaks, and using synthetic FAC, *N*-linolenoyl-L-Gln as calibration standard. “nd” refers to samples with no FAC detected. Data (n = 3) are shown as means ± SD.

**Figure 3:**
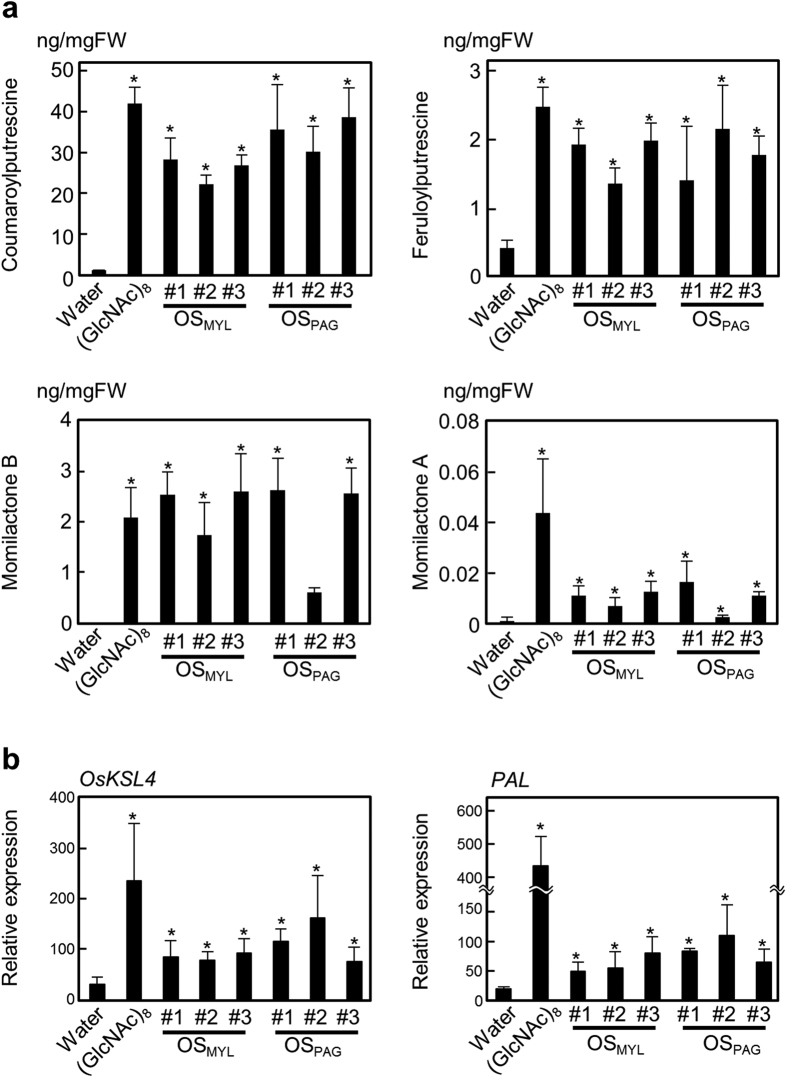
OS from *M. loreyi* and *P. guttata* elicits defense metabolic responses in rice cells. Freshly prepared aliquots of rice cells in 1 mL culture media were treated with 2 μL OS from *M. loreyi* (OS_MYL_) or *P. guttata* (OS_PAG_) for 24 h (i.e., final 500-fold OS dilution). Treatment with fungal elicitor (GlcNAc)_8_ at 10 nM concentration was included as positive control in all experiments. Metabolite accumulation (**a**) and gene expression levels (**b**) were determined 24 h and 1 h after elicitation, respectively. Data (n = 3–4) are shown as means ± SD, and asterisks show statistically significant differences between individual elicitor treatments and control (water) analyzed by Dunnett’s test (*P < 0.05).

Oral secretions from *M. loreyi* (OS_MYL_) and *P. guttata* (OS_PAG_) (Fig. 2a) collected from 4–5^th^ instar larvae were examined by LC-MS method for their respective FAC contents. At least four typical FACs, *N*-hydroxylinolenoyl-L-Gln, *N*-hydroxylinoleoyl-L-Gln, *N*-linolenoyl-L-Gln and *N*-linoleoyl-L-Gln were detected in OS_MYL_ but no typical FAC was found in the OS_PAG_ (Fig. 2b). Using synthetic *N*-linolenoyl-L-Gln as calibration standard, cumulative 0.1–0.7 mM concentrations of FACs in the independent OS_MYL_ isolates were estimated (Fig. 2c). Considering the substantial variation in the FAC contents among individual OS isolates, several batches of OS_MYL_ and OS_PAG_ collected on different days were subsequently used to treat rice cells divided in 40 mg aliquots. Chitin octamer ((GlcNAc)_8_), fungal elicitor known to strongly elicit defense responses in rice cells was used as a positive control to observe the OS_MYL_ and OS_PAG_ elicitor activities^34^,^39^,^40^. Compared to mock treatment at 24 h, all metabolic markers of defense (CoP, FP, MoA and MoB) accumulated significantly more after treatment of rice cells with OS_MYL_ and OS_PAG_, and (GlcNAc)_8_ (Fig. 3a). At transcript levels, the expression of rice *KAURENE SYNTHASE-LIKE 4* (*OsKSL4*) gene essential for momilactone biosynthesis^41^, and *PHENYLALANINE AMMONIA-LYASE* (*OsPAL*) required for phenolamide production^21^ were both significantly higher in OS_MYL_, OS_PAG_, and (GlcNAc)_8_ treatments compared to mock (Fig. 3b), collating with the observed metabolite accumulation patterns (Fig. 3a). Furthermore, both OS types and chitin promoted the accumulation of jasmonates, jasmonic acid (JA) and jasmonoyl-L-isoleucine (JA-Ile) in rice cells (Supplemental material Figure S1) that was likely involved in the regulation of these metabolic changes.

**Figure 4:**
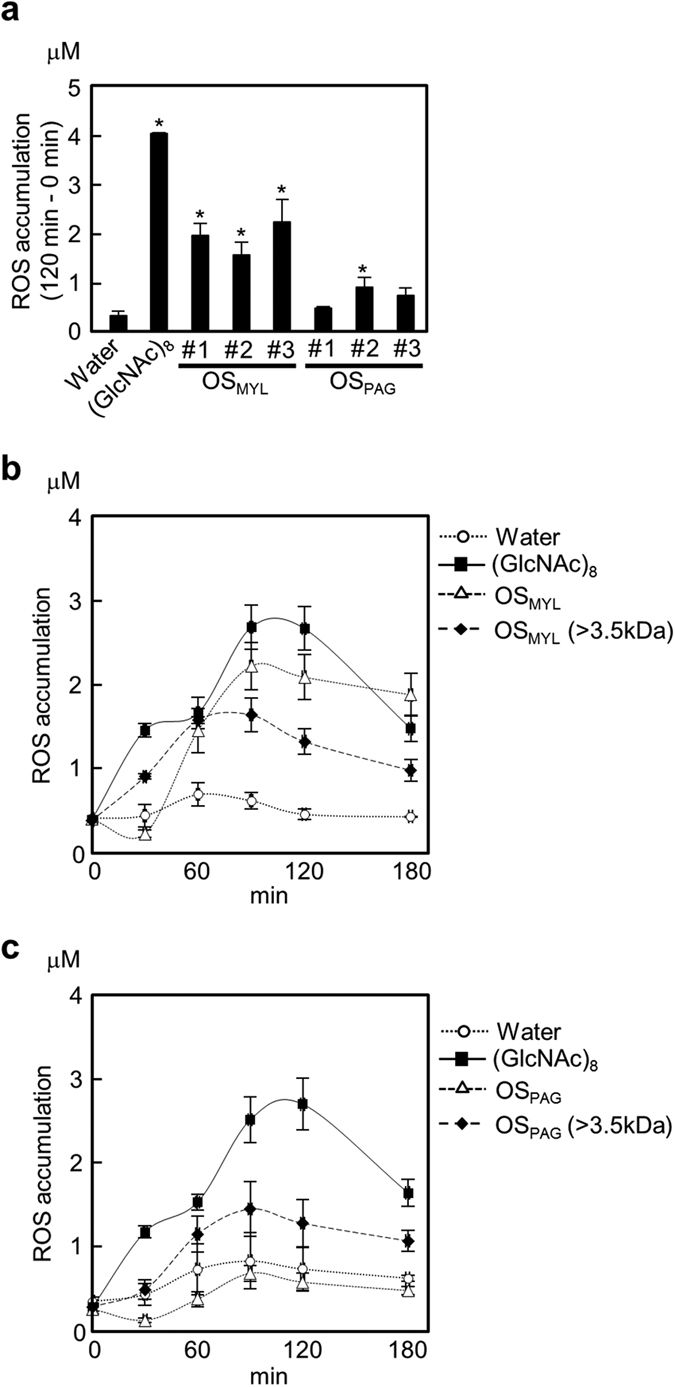
OS_MYL_ and OS_PAG_ induce ROS burst in rice cells. Rice cells were treated with OS as before, and accumulation of ROS in culture media was determined by chemiluminescence assays with L-012 as substrate. (**a**) ROS accumulations captured 120 min after OS application (each OS_MYL_ and OS_PAG_ belong to independent batch of larvae #1–3). (**b**,**c**) Time resolved course of ROS accumulation elicited by crude OS (500-fold dilution) or OS dialysate (>3.5 kDa, 500-fold dilution), carefully adjusted to comparable amounts in both experiments. Data (n = 3) are shown as means ± SD, and asterisks show statistically significant differences between treatments and control determined by Dunnett’s test (*P < 0.05).

We next examined the OS-induced release of ROS, which is another potential intermediate signal for activation of plant defense. Interestingly, ROS accumulation curves induced by OS_MYL_ and OS_PAG_ in rice cells had a single phase, while chitin octamer induced two-phased ROS burst^34^. In addition, OS_MYL_ induced strong ROS burst but crude OS_PAG_ showed much weaker activity on ROS (Fig. 4a–c). At least in part, this could be attributed to the substantial inhibitor-like activity on ROS contained in OS_PAG_ that was considerably diminished by dialyzing the crude OS_PAG_ samples over the 3.5 kDa cutoff membrane (Fig. 4c). Similar, but less pronounced inhibition was also observed in OS_MYL_, where the ROS levels initially appeared lower than water-treated controls (compare early 30 min time point in Fig. 4b, Fig. S2). Notably, dialysis and removal of inhibitor activity from OS_PAG_ resulted in ROS accumulation patterns that were comparable to OS_MYL_ (compare dialysate-treated cell ROS levels in Fig. 4b,c). Although OS_MYL_ dialysis could restore full ROS response at the early time points, the overall activity of dialysate remained lower (see Fig. 4b; 90–180 min), suggesting the loss of a putative <3.5 kDa elicitor component from the OS_MYL_ after dialysis. This loss could be later assigned to the small molecular FACs found abundantly in OS_MYL_ (Fig. 2b,c) as described in the following text. Next, we focused on the possible molecular interactions between FACs and HME(s) contained in the OS-isolated fraction.

### FAC potentiates rice cell responses to HME fraction

**Figure 5:**
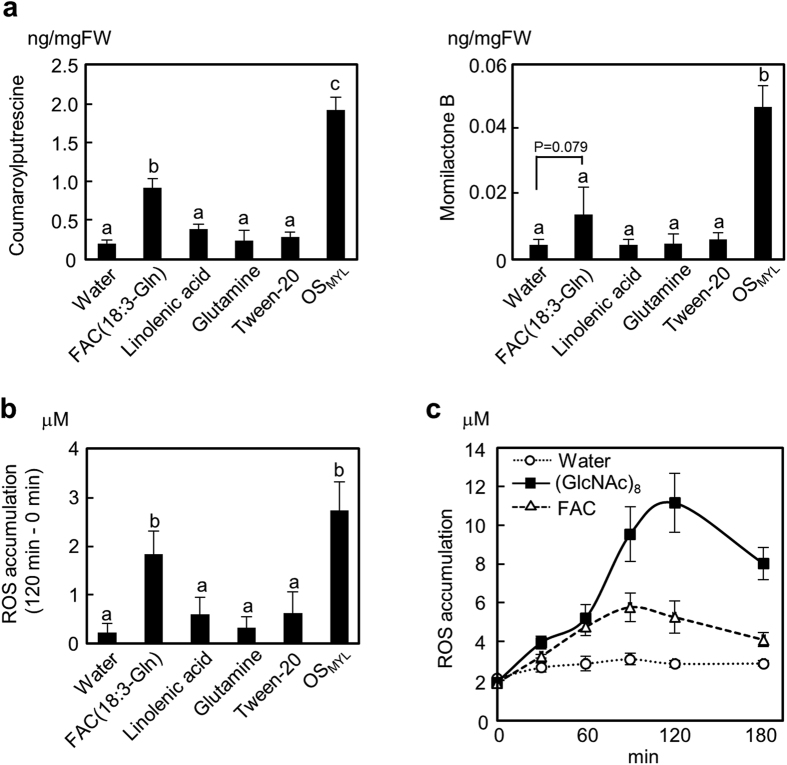
Activity of synthetic *N*-linolenoyl-L-Gln in rice cells. Rice cells were treated with synthetic *N*-linolenoyl-L-Gln (50 μM), linolenic acid (50 μM), Gln (50 μM), Tween-20 [0.01% (v/v)], OS_MYL_ (500-fold dilution), or (GlcNAc)_8_ (10 nM). Stock solutions of *N*-linolenoyl-L-Gln, linolenic acid, and Gln were dissolved in 0.01% (v/v) Tween-20 that was included as one of negative control treatments. (**a**) Metabolite accumulation measured 24 h after elicitation. (**b**) ROS amounts captured 120 min after elicitation. (**c**) Time resolved ROS release 0–180 min after FAC and (GlcNAc)_8_ treatments. Data (n = 3) are shown as means ± SD, and statistical differences were analyzed by ANOVA followed by Tukey HSD test (*P* < 0.05).

**Figure 6:**
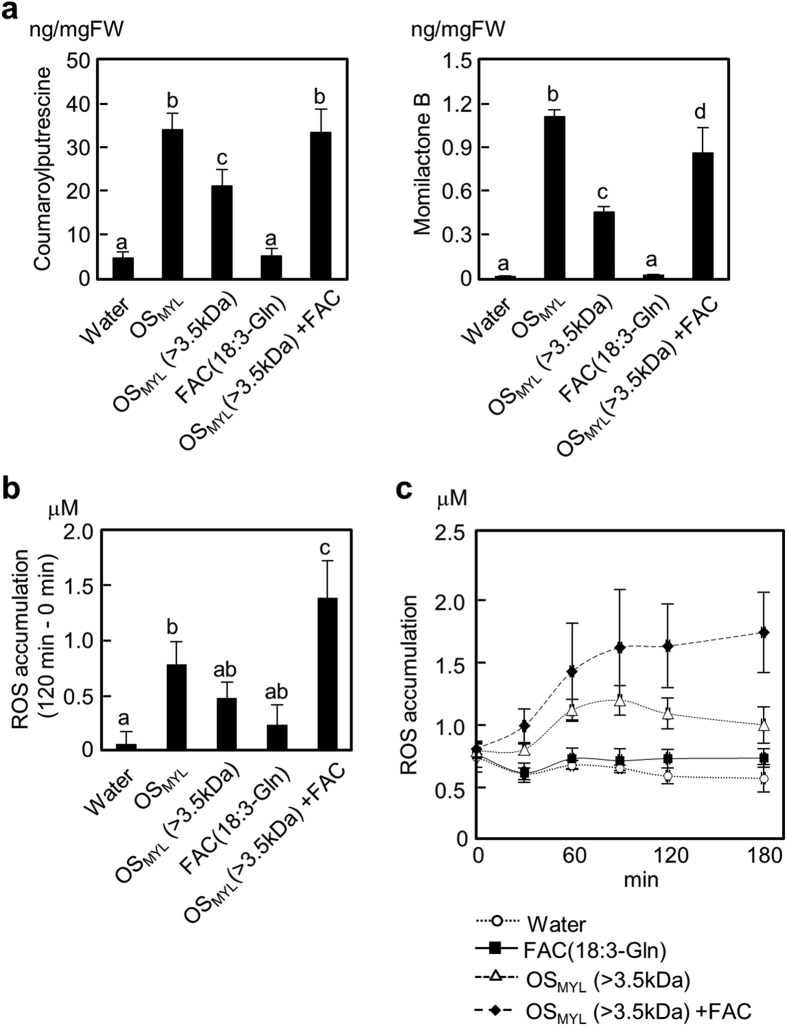
Synergistic action of FAC and HME fraction from OS_MYL_. Metabolite accumulation was measured 24 h after OS_MYL_ (500-fold dilution), OS_MYL_ dialysate (>3.5 k Da, 500-fold dilution; HME fraction) and FAC (*N*-linolenoyl-L-Gln, 10 μM) application to freshly prepared aliquots of rice cells (amounts of crude OS_MYL_ and OS_MYL_ dialysate were carefully adjusted to comparable levels in each experiment). Data (n = 3) are shown as means ± SD. (**b**) ROS amounts captured 120 min after treatment. (**c**) Time resolved ROS release 0–180 min after elicitation. Data (n = 3) are shown as means ± SD, and statistical differences were analyzed by ANOVA followed by Tukey HSD test (*P* < 0.05).

The elicitor activity of FACs in wounded plants was previously reported^42^; however, the activity of FAC in rice was not yet examined. Here, we analyzed the activity of 50 μM synthetic *N*-linolenoyl-L-Gln, which is the most abundant OS_MYL_ FAC (Fig. 2a) in rice cell system. The high concentration of *N*-linolenoyl-L-Gln significantly promoted CoP and MoB accumulations, and initiated single-phased curve of ROS accumulation in rice cells (Fig. 5a–c). As expected, free linoleic acid or Gln did not show any elicitor activity at similar concentrations. However, compared to 50 μM *N*-linolenoyl-L-Gln, much lower concentrations of FAC (2 μM or 10 μM) showed little effect on metabolites in rice cells (Fig. 6 and Supplemental material Figure S3).

In our typical experimental setup, only 2 μL of OS_MYL_ were applied to 1 mL culture media, resulting in 1/500 OS_MYL_ dilution factor, and thus ~2 μM final concentration of FACs in solution (Figs 2 and 3). Notably, this was still sufficient to elicit rice responses because the removal of FACs by dialysis (>90%) resulted in approximately 30% and 50% lower contents of CoP and MoB in rice cells, respectively (Fig. 6). This suggested that interaction of some small molecular component(s), such as FACs, with the HME fraction, rather than small molecules alone modulated the OS_MYL_ responses. We then applied 10 μM synthetic *N*-linolenoyl-L-Gln together with the HME fraction to rice cells to test this hypothesis.

**Figure 7:**
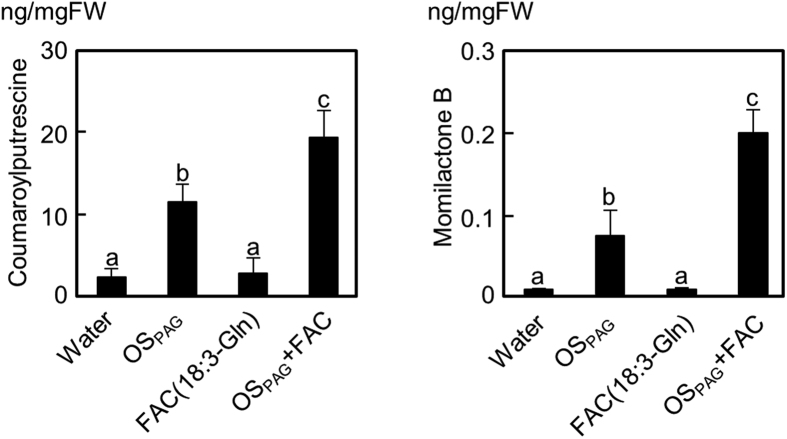
Synthetic FAC enhances OS_PAG_ -induced defense responses. CoP and momilactone B accumulations were determined 24 h after OS_PAG_ (500-fold dilution), FAC (*N*-linolenoyl-L-Gln, 10 μM), or both elicitors were added to freshly prepared aliquots of rice cells. Data (n = 3) are shown as means ± SD, and statistical differences were analyzed by ANOVA followed by Tukey HSD test (*P* < 0.05).

As anticipated, *N*-linolenoyl-L-Gln significantly promoted CoP and MoB accumulations compared to treatment with the HME fraction alone. In addition, ROS generation in rice cells was also promoted by the addition of as low as 10 μM *N*-linolenoyl-L-Gln to the HME fraction (Fig. 6b). In all cases, two-way ANOVA analysis indicated statistically significant (*P* < 0.05) synergistic interactions between the HME fraction and FAC elicitors on rice cells. Finally, although original OS_PAG_ did not contain any FACs (Fig. 4), OS_PAG_-dependent accumulation of CoP and MoB was enhanced by the addition of 10 μM *N*-linolenoyl-L-Gln (Fig. 7; two-way ANOVA synergistic interaction, *P* < 0.05), proposing a function for FACs, and possibly other small elicitor molecules, as enhancers of HME fraction-induced plant defense.

## Discussion

Identification of new insect elicitors and understanding their molecular interactions are essential for dissecting the non-self-recognition signaling cascades in plants. Here, we show an elicitor fraction from the *M. loreyi* OS, which is different from the conventional FACs or other elicitors from insects effective in plants^7^. Because signaling of this elicitor fraction is promoted by FACs, it provides the first well-documented example of positive interaction between the two naturally co-existing insect elicitors in plant defense.

### Cell approach to study perception of herbivory in plants

Insect elicitors are well known modulators of plant defense responses. For example, plants can emit differential blends of VOCs in response to different herbivores, or developmental stage of the same herbivore (reviewed in ref. 17) but perception and regulatory mechanisms leading to such differences remain largely unknown. As repeated mechanical wounding already induced plant defense responses similar to herbivory in Lima beans (*Phaseolus lunatus* L.)^1^, it suggests that in the real world, various signals are integrated during herbivore attack, such as HAEs and repeated character of mechanical stress (possibly in association with DAMPs and others), to activate specific defense responses against various types of herbivores. While decoding contribution of each individual factor at molecular level is important, separation of the wound/DAMPs and elicitor effects remains a significant challenge in the intact plants.

Here, rice cells provide good alternative for rapid detection and characterization of responses to elicitors from insects that is however at the expense of the natural spatio-temporal processes occurring during caterpillar feeding on intact plants. Previously, cell approach was used for functional analyses of microbial elicitors in rice^35^,^43^,^44^,^45^. As main advantage, aliquots of rice cells can be prepared with high reproducibility, allowing higher throughput and low experimental variation, and crude or partially purified insect elicitors can be directly applied to the cells without additional wound stress. Importantly, isolated cells and intact plant tissues share very similar defense profiles at the metabolic level. For example, tobacco and periwinkle (*Catharanthus roseus*) cell cultures treated with MeJA (volatile form of JA), and rice cells treated with JA produced large amounts of secondary metabolites that were also found in the intact plants^21^,^46^,^47^. Non-green rice cells used in our study accumulated high levels of PAs, CoP and FP, known to act in defense against sucking insects^23^. Although we could not yet demonstrate the efficacy of phenolamides against rice chewing herbivores, by supplying them in artificial diet to *M. lorey*i or *S. mauritia* larvae, previous work with tobacco silenced in the expression of the master phenolamide regulator MYB8 showed that PAs are important players in plant defense against leaf chewers^22^. We are now cloning and silencing the rice equivalent(s) of MYB8 to demonstrate additional functions of PAs in rice defense.

### Rice plant-herbivore interface

A direct comparison of the OS_MYL_ and OS_PAG_ suggested that rice defense depends on multiple signals in the OS that converge into one or more defense signaling pathways. Even without FACs in *P. guttata* OS, rice cells could still effectively respond to this OS. Notably, OS_PAG_ strongly suppressed ROS released from rice cells by yet unidentified small size inhibitor (Fig. 4). This inhibitor, however, did not affect substantially the OS_PAG_-elicited CoP metabolic responses in rice cells (Fig. 3). It suggests that PA defense responses might be, largely or completely, independent of ROS signaling. However, ROS signaling could be involved in other independent defense responses that were not addressed in this work, such as the VOC emissions and indirect defenses against herbivores in rice.

In this work, the role of FACs in rice was distinctly different from the dominant role of FACs described in *M. sexta*-tobacco interactions^16^. OS_MYL_ retained relatively high elicitor activity in rice cells even after removing the majority of its FACs by dialysis. A similar observation was also reported in the *N. attenuata* - *Spodoptera littoralis* interactions^48^; the FAC-free oral secretions after ion-exchange column chromatography still significantly enhanced the JA accumulation in an FAC-independent manner.

In our current view of plant-herbivore interface, non-FAC elicitors can be proposed as an important alternative and/or supplement to FACs during perception of herbivory in plants.

### HME fraction from insect OS

While probing FACs as major elicitors in rice plants, predominant elicitor activity was surprisingly associated with the high molecular mass OS fraction obtained by dialysis of OS_MYL_ and OS_PAG_ (3.5 kDa cutoff). Several high molecular mass herbivore-associated elicitors have already been reported. Namely, porin-like protein and β-glucosidase enzyme are two proteinous type elicitors isolated from herbivores^12^,^49^. However, the HME fraction-contained elicitor(s) was not an enzyme as it was not affected by heat treatment after dialysis (see Materials and Methods). Recently, an HME fraction active in Arabidopsis was isolated by gel filtration from *S. littoralis* OS^50^. Interestingly, this fraction contained a putative β-galactofuranose polysaccharide of unknown detailed structure. Future comparison of HME fraction components from rice herbivores and this elicitor will be of the prime interest. Resolving complex polysaccharide structures and their elicitor activities in plants remain our ongoing challenges.

### Synergism in insect elicitor action

We could demonstrate a previously unnoticed interaction between the HME fraction-contained elicitor(s) isolated in this study, and canonical FACs. While the synthetic FAC alone was almost inactive in rice cells at concentrations present in applied OS (Fig. 6 and Fig. S3), it strongly enhanced defense responses induced by HME fraction from OS_MYL_ and crude OS_PAG_. This suggests that in rice, and possibly other plants, FACs may function as amplifiers of basal defense responses modulated by the HME fraction-contained elicitor(s). Amplification and/or synergistic action of two elicitors has already been reported in MAMP signaling^35^,^51^,^52^. For example, simultaneous application of fungal elicitor (GlcNAc)_8_ and lipopolysaccharide (LPS) enhanced defense responses in rice cells, even at very low concentrations of bacterial LPS. Similar enhancement of defense responses was documented in rice seedlings, supporting the physiological relevance of results obtained in cell-based systems^35^.

Hence, plants sense microbial infections thorough multiple elicitors, including MAMPs and DAMPs. It is not then surprising that elicitor crosstalk has also evolved in plant-insect interactions and herbivory that originated ca. 400 Ma ago^53^. Simultaneous recognition of various elicitors and stimuli^4^ is likely to make plant defenses more robust and durable, even if some insects have tried to escape the detection by use of various effectors against both specific and general plant signaling components, such as receptors and/or signal transduction pathways^6^,^7^.

### Future of molecular plant-insect interactions

Plant defense against herbivores emerges as complex process driven by evolution of species-specific interactions, differences between generalist and specialist herbivores, variable feeding strategies of insects, and defense strategies adopted by plants. In such molecular maze, many important signals remain well hidden at the plant-insect interface, such as many OS components that interact with disrupted and/or intact plant cells. The interaction of FACs with HME as we show it here is likely just a tip of an iceberg. Further identification of insect elicitors and effectors, their receptors and signaling pathways, and their mutual interactions are necessary to build a stronger foundation for the understanding of plant-herbivore interactions at molecular level.

## Materials and Methods

### Plant materials and treatments

Rice plants (*Oryza sativa* var. Nipponbare) were germinated in nutrient-rich soil pellets Kumiai Ube Baido No.2 (MC Ferticom, Tokyo, Japan). After 10 days, plantlets were transferred to larger pots with paddy field soil partially supplemented with nutrient-rich pellets (~20%). Experiments were conducted with 4–6 week old plants using the youngest fully developed leaf (~20 × 1 cm). For mimic herbivory treatments, the youngest fully developed leaf on each seedling was wounded with a fabric pattern wheel along the midvein, and wounds were immediately treated with 20 μL of water or 20 μL of water-diluted [1:3 (v/v)] oral secretions (OS). Direct feeding was conducted with 2–3^rd^ instar larvae attached to the youngest fully developed leaf in a clip cage^23^. Non-green rice suspension cell cultures (*Oryza sativa* L. cv. Nipponbare) were derived from mature rice seeds with embryos placed on the modified N6 callus-forming agar media supplemented with 1 mg/L 2,4-D. The cells were transferred to 300 mL conical flasks and propagated weakly in suspension in modified N6 culture medium as described previously^34^. Typically, 40 mg aliquots of rice cells at 4^th^ day after subculture were used in bioassays.

### Insects and collection of oral secretions

Larvae of Loreyi armyworm *Mythimna (Leucania) loreyi* (MYL; generalist) and rice skipper *Parnara guttata* (PAG; specialist) were collected in paddy field in Kurashiki (Okayama prefecture, Japan), and reared under laboratory conditions on artificial pinto bean-based diet (generalist MYL) or rice leaf (specialist PAG). Oral secretions (OS_MYL_ and OS_PAG_) were collected from larvae exclusively fed on rice leaves at least 2–3 d prior to OS collection. Larvae were held between fingertips and mechanically disturbed with blunted micropipette tip (200 μL) directly connected to polypropylene tubing maintained under mild vacuum. OS accumulated in a vacuum tubing-interconnected 2 mL collection tube that was kept on ice to prevent OS degradation. All OS fractions were kept in original isolation batches and stored frozen at −80 °C until needed. Before use, each OS batch was centrifuged at 14,000 *g* (4 °C) and supernatants were used for elicitor bioassays, OS chemical analyses, and elicitor purifications. To obtain the high molecular mass fraction from OS_MYL_ and OS_PAG_, OS samples were dialyzed against pure water for 2 days at 4 °C in a 3,500 molecular weight cutoff dialysis tubing (BioDesign Inc.). Dialysates were heated at 95 °C for 5 min and subsequently centrifuged at 14,000 *g*. Supernatants were used for elicitor bioassay after necessary dilutions essentially as described in text.

### Determination of FACs in OS

The OS isolates were centrifuged and diluted 100-fold before applying 10 μL on a triple quadrupole LC-MS/MS 6410 system (Agilent Technologies, Santa Clara, CA, USA) equipped with a Zorbax SB-C18 column [50 × 2.1 mm ID, 1.8 μm, Agilent Technologies]. MS was set to operate in negative electrospray ionization (ESI) mode and mass scan data were collected in *m/z* 150–1000 range. Sample separation was achieved with solvent A [0.1% (v/v) formic acid in water] and solvent B [0.1% (v/v) formic acid in acetonitrile] in time (min)/B (%) gradient program: 0/5, 0.5/5, 2/40, 6/40, 10/95, 15/95, 16/5, 20/5. Constant flow rate of 0.4 mL/min was applied to chromatographic column placed in thermostatic chamber controlled at 40 °C. Additional MS variable parameters were used as follows: fragmentor 135V, dwell 200, and 7V cell accelerator voltage. Synthetic FAC (*N*-linolenoyl-L-Gln) was used to estimate FAC content in individual OS isolates, which was then expressed as *N*-linolenoyl-L-Gln equivalents.

### Analysis of rice metabolic responses

The rice cells (40 mg) were placed in 24-well microtiter plate and pre-incubated in 1 mL fresh culture media for 30 min to subdue initial stress-induced ROS levels. Equivalent amounts of elicitors and water were applied to treatment and mock control cell groups, respectively. Chitin oligomer (GlcNAc)_8_ was used as positive control treatment at 10 nM concentration^35^. ROS released from rice cells into culture medium was quantified by previously established chemiluminescence method with sensitive L-012 substrate^54^,^55^. Chemiluminescence was detected in microplate luminescence reader (PowerScan HT, DS Pharma Biomedical) in 30–60 min time intervals up to 3 h. The amount of ROS was calculated against authentic calibration curves prepared as serial dilutions of hydrogen peroxide (H_2_O_2_) standard in each experiment. To test a possible direct inhibitory effect of OS_MYL_ and OS_PAG_ on the chemiluminescence assays (e.g., quenching), OS_MYL_ and OS_PAG_ (final 500-fold dilution) were mixed with hydrogen peroxide solution (final conc.10 μM) in a media without cells, and chemiluminescence assay was carried out as before (Supplemental material Figure S3).

### Quantitative RT-PCR

To quantify gene expression, qRT-PCR was performed essentially as described^56^ using authentic calibration curves prepared from serially diluted cDNA samples. Total RNA samples were prepared from rice cells (40 mg) using TRIzol reagent according to the manufacturer’s protocol (Invitrogen, Carlsbad, CA, USA). The cDNA was synthesized by PrimeScript (Takara Bio Inc., Japan) reverse transcriptase enzyme after completing standard DNase treatment and subsequent cleanup of RNA samples. Transcript levels were detected by THUNDERBIRD qPCR Mix (Toyobo, Osaka, Japan) on a 7500 Real-Time PCR system (Applied Biosystems, Foster City, CA, USA). *OsEF1α* gene was used as an internal control to correct for differences in RNA amounts or sample quality amongst individual RNA preps. Primer sequences used for qRT-PCR were (forward/reverse): *PAL* (5-CTACCCGCTGATGAAGAAGC-3/5-GCACCTTGTTCAGCTCCTCG-3), *OsKSL4* (5-CGGTGTCATTCCTAAATCATGCAAGG-3/5-CGGCCTGAGAGTAGAACACA-3)^57^. Housekeeping gene primers were specific to *OsEF1α* (5-CTGCCACACCTCCCACATTGC-3/5-CCGCACGGCAAAACGACCA-3).

### Quantification of defense metabolites

Metabolites were extracted from rice cells (40 mg) or rice leaves (80–100 mg) and measured on a triple quadrupole LC-MS/MS 6410 system (Agilent Technologies, Santa Clara, CA, USA) equipped with a Zorbax SB-C18 column (50 × 2.1 mm ID, 1.8 μm, Agilent Technologies) as described previously^23^. Amount of each metabolite was estimated using synthetic standards (CoP, FP) or purified chemicals momilactone A (MoA) and momilactone B (MoB).

### Elicitor compounds

(GlcNAc)_8_ was prepared from chitosan oligosaccharide, (GlcN)_8_, by acetylation^58^. *N*-linolenoyl-L-Gln was prepared from linolenic acid and Gln as described previously^59^, and modified as described below. A solution of 1227 uL (1122 mg, 4 mmol) linolenic acid, 669 uL (4.8 mmol) triethylamine in 25 mL freshly distilled tetrahydrofuran (THF) at 0 °C was stirred and 535 uL (4.34 mmol) pivaloyl chloride (trimethyl acetyl chloride) was added, and stirring continued for 2 h. The solution was filtered quickly and the white cake washed with 25 mL THF. The filtrate and washings, containing the FA anhydride, were combined, diluted with 25 mL 1,4-dioxane and used as acylating reagent in following reaction. To a solution of 1115 mg (8 mmol) of glutamine in 6.4 mL water (not fully dissolved) 1114 uL (8 mmol) trimethylamine was added, stirred at RT for 2 h and the acylating reagent added at RT and stirred. After 20 min, 562 uL (4.03 mmol) trimethylamine was added and allowed to stand overnight. The white granular solids in flask were adjusted the pH 3 with 5 mL 1 M HCl. The precipitate dissolved and the solution was a single phase. Sequentially, 50 mL water and then 50 mL dichloromethane (DCM) were added to achieve phase separation (lower: upper 3:1 by volume). The upper phase was re-extracted with 50 mL DCM then 20 mL DCM. The DCM extracts were combined and washed with 100 mL NaCl-saturated water (Brine, 345 g/L, SG 1.22). Brine is heavier than the DCM extracts (DCM SG 1.33) because of the presence of THF and dioxane in the latter phase. The DCM phase was dried with MgSO_4_ and evaporated to dryness in the presence of 10 g silica. The 10 g silica/reaction products, as DCM slurry were applied to a column of 30 g silica and 12.5 mL fractions were eluted with 150 mL DCM, then 125 mL DCM: methanol 4:1 and finally with 150 mL DCM: methanol 2:1. Each fraction (1 uL) was run on TLC using DCM: methanol 2:1 as solvent. The major product eluted in fractions 16–19, soon after the switch to DCM: methanol 2:1. Fraction 19 (cleanest) was analyzed by MS and shown to be expected product. The final product (1.1 g) after evaporation of the four fractions combined was a waxy pale yellow solid that completely dissolved in 0.5 mL acetonitrile. At 4 °C, the yellow solution went solid. N.B. this compound is volicitin without the 17-hydroxy on the fatty acid.

### Statistical analyses

Statistical analyses (one, two-way ANOVA) were carried out with an open source software OpenStat (http://statpages.info/miller/OpenStatMain.htm) or commercial version of Microsoft Excel (Student’s t-test). Multiple comparisons where each experimental mean was compared with the control mean were analyzed by Dunnett’s test (http://www.gen-info.osaka-u.ac.jp/MEPHAS/dunnett-e.html) after normality test by Shapiro-Wilks method (OpenStat). Data showing deviation from normal distribution were log_2_ transformed before statistical analysis.

## Additional Information

**How to cite this article**: Shinya, T. *et al*. Modulation of plant defense responses to herbivores by simultaneous recognition of different herbivore-associated elicitors in rice. *Sci. Rep.* **6**, 32537; doi: 10.1038/srep32537 (2016).

## Supplementary Material

Supplementary Information
