# Supplementary material for: Modulation of plant defense responses to herbivores by simultaneous recognition of different herbivore-associated elicitors in rice
Source: Sci Rep. 2016 Sep 1;6:32537. doi: 10.1038/srep32537 (PMC5007475; doi:10.1038/srep32537)
Supplement: Supplementary Information [file srep32537-s1.pdf]

Supplementary information

Modulation of plant defense responses to herbivores by simultaneous recognition of different herbivore-associated elicitors in rice

Tomonori Shinya, Yuko Hojo, Yoshitake Desaki, John T. Christeller, Kazunori Okada, Naoto Shibuya, and Ivan Galis

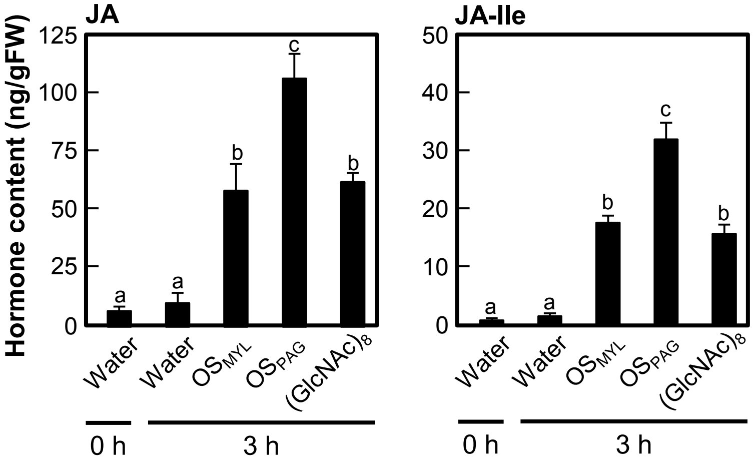

Supplemental Figure S1

Phytohormone levels in elicitor treated rice cells

Effect of OS_{MYL} (100-fold dilution), OS_{SKP} (100-fold dilution) and chitin ($(GlcNAc)_8$, 100 nM) on jasmonate levels 3h post treatment was determined by LC-MS/MS as described previously[1]. Data (n= 4) are shown as means ± SD, and statistical differences were analyzed by ANOVA followed by Tukey HSD test ($P < 0.05$).

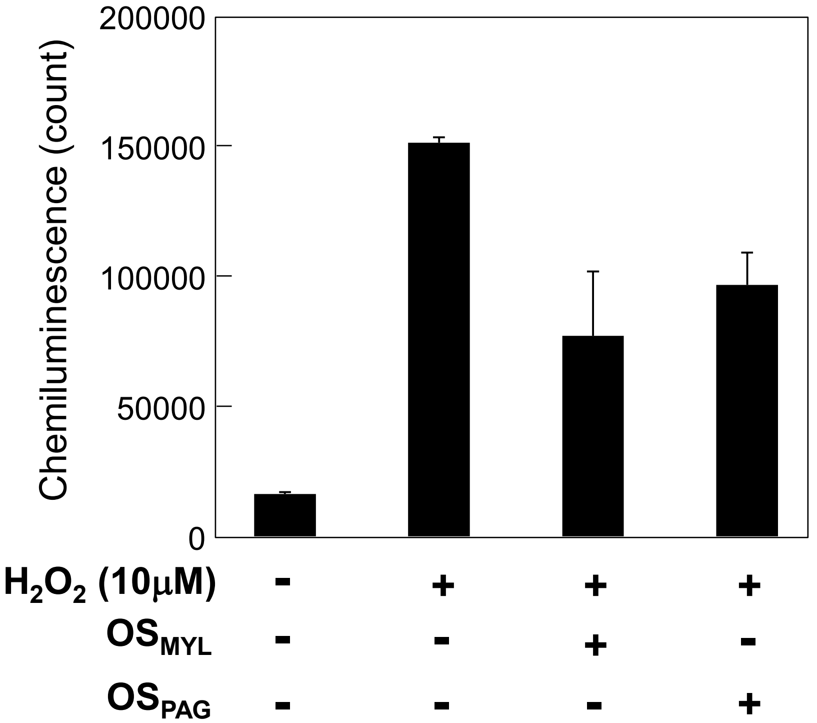

Supplemental Figure S2

Effect of OS_{MYL} and OS_{PAG} on chemiluminescence assay

Effect of OS_{MYL} and OS_{PAG} on chemiluminescence assay was determined using three independent batches of oral secretions. Data (n= 3) are shown as means ± SD.

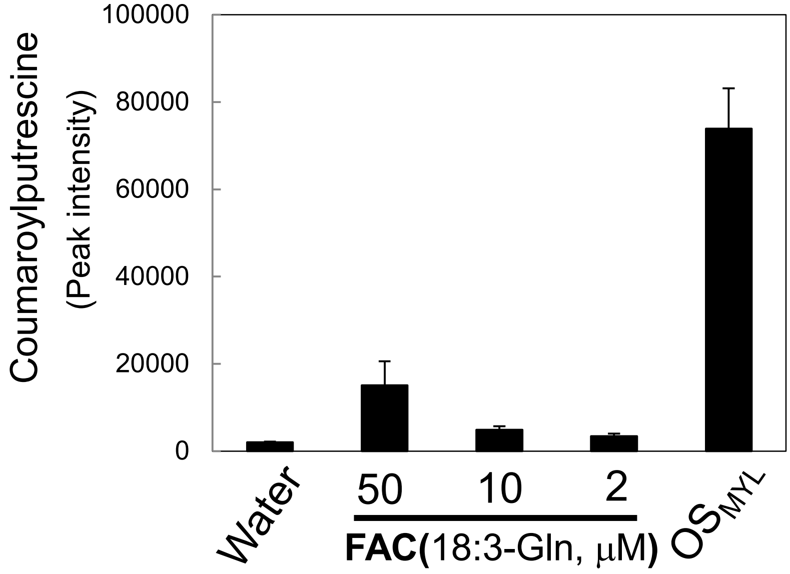

Supplemental Figure S3

Elicitor activity of synthetic *N*-linolenoyl-L-Gln in rice cells

Rice cells were treated with various concentrations of synthetic FAC, *N*-linolenoyl-L-Gln (18:3-Gln), or OS_{MYL} at 500-fold final dilution. CoP accumulation was measured at 24 h after cell elicitation. Data (n= 3) are shown as means ± SE.

Supplemental section reference:

1 Fukumoto, K. *et al.* Response of rice to insect elicitors and the role of OsJAR1 in wound and herbivory-induced JA-Ile accumulation. *J. Integr. Plant Biol.* **55**, 775-784 (2013).
